# Topical Ascorbic Acid Ameliorates Oxidative Stress-Induced Corneal Endothelial Damage via Suppression of Apoptosis and Autophagic Flux Blockage

**DOI:** 10.3390/cells9040943

**Published:** 2020-04-11

**Authors:** Yi-Jen Hsueh, Yaa-Jyuhn James Meir, Lung-Kun Yeh, Tze-Kai Wang, Chieh-Cheng Huang, Tsai-Te Lu, Chao-Min Cheng, Wei-Chi Wu, Hung-Chi Chen

**Affiliations:** 1 Department of Ophthalmology, Chang Gung Memorial Hospital, Linkou branch, Taoyuan 33305, Taiwan; 2 Center for Tissue Engineering, Chang Gung Memorial Hospital, Linkou branch, Taoyuan 33305, Taiwan; 3 Department of Biomedical Sciences, Chang Gung University College of Medicine, Taoyuan 33305, Taiwan; 4 Department of Medicine, Chang Gung University College of Medicine, Taoyuan 33305, Taiwan; 5 Institute of Biomedical Engineering, National Tsing Hua University, Hsinchu 30012, Taiwan

**Keywords:** ascorbic acid, oxidative stress, apoptosis, autophagic flux blockage, PI3K/Akt, corneal endothelial cells

## Abstract

Compromised pumping function of the corneal endothelium, due to loss of endothelial cells, results in corneal edema and subsequent visual problems. Clinically and experimentally, oxidative stress may cause corneal endothelial decompensation after phacoemulsification. Additionally, in vitro and animal studies have demonstrated the protective effects of intraoperative infusion of ascorbic acid (AA). Here, we established a paraquat-induced cell damage model, in which paraquat induced reactive oxygen species (ROS) production and apoptosis in the B4G12 and ARPE-19 cell lines. We demonstrate that oxidative stress triggered autophagic flux blockage in corneal endothelial cells and that addition of AA ameliorated such oxidative damage. We also demonstrate the downregulation of Akt phosphorylation in response to oxidative stress. Pretreatment with ascorbic acid reduced the downregulation of Akt phosphorylation, while inhibition of the PI3K/Akt pathway attenuated the protective effects of AA. Further, we establish an in vivo rabbit model of corneal endothelial damage, in which an intracameral infusion of paraquat caused corneal opacity. Administration of AA via topical application increased its concentration in the corneal stroma and reduced oxidative stress in the corneal endothelium, thereby promoting corneal clarity. Our findings indicate a perioperative strategy of topical AA administration to prevent oxidative stress-induced damage, particularly for those with vulnerable corneal endothelia.

## 1. Introduction

Phacoemulsification is the most commonly practiced surgery for the treatment of cataracts. Complications of phacoemulsification that compromise visual prognosis remain a concern. These include cystoid macular edema [1], infectious endophthalmitis [2], retinal detachment [3], and corneal edema due to loss of human corneal endothelial cells (HCECs) [4]. The overall rate of HCEC loss after cataract surgery is 2.5% annually [5], while rates of 5.1% [6] to 12.1% [7] have been reported among patients with low HCEC density. HCECs play a cardinal role in the regulation of stromal hydration and corneal transparency [8]. HCECs have been found to exhibit limited proliferative potential, both clinically [9,10] and experimentally [11,12]. We, therefore, wondered whether there were effective and safe strategies to prevent loss of HCECs during and after phacoemulsification.

The current knowledge of the causative mechanisms underlying changes in HCECs following phacoemulsification is insufficient. A clinical study observed that reactive oxygen species (ROS) or free radicals generated by high-intensity ultrasound oscillations in water during phacoemulsification damaged the corneal endothelium [13]. Such phenomena have also been demonstrated in vivo [14,15] and in vitro [16,17]. Furthermore, significant induction of apoptosis has been reported in experimental studies of corneal endothelial cells during phacoemulsification [15,16,18]. Oxidative stress has been reported to induce autophagosome formation [19] in various ocular [20] and systemic [21] diseases. However, until now, there have been no reports demonstrating autophagic flux blockage in ROS-induced corneal endothelial damage. 

Conventionally, ascorbic acid is considered an ideal ocular nutritional supplement [22]. Evidence has shown that ascorbic acid inhibits apoptosis of murine [23,24], bovine [16], and human corneal endothelial cells [25], probably through protection against oxidative stress and damage. Likewise, evidence from studies on rabbit eyes has also suggested a protective effect of intraoperative infusion of ascorbic acid during phacoemulsification, due to either free-radical-scavenging properties [14,26] or oxidative stress reduction [15]. Nevertheless, there have been no previous studies focusing on the application of topical ascorbic acid for the corneal endothelium to prevent oxidative stress generated during phacoemulsification.

In this study, we aim to investigate the protective effect of ascorbic acid against oxidative stress in HCEC. Our findings support the hypothesis that ascorbic acid attenuates oxidative stress in HCECs through inhibition of apoptosis and autophagic flux blockage. Furthermore, in a rabbit model, we demonstrate that topical administration of ascorbic acid ameliorates corneal endothelial damage caused by oxidative stress through increasing the concentration of ascorbic acid in the corneal stroma.

## 2. Materials and Methods

### 2.1. Materials

Cell culture media and various additives, comprising Dulbecco modified Eagle’s medium (DMEM), DMEM/F12, Opti-MEM, human endothelium serum-free medium (HESFM), trypsin-EDTA, fetal bovine serum (FBS), phosphate-buffered saline (PBS), gentamicin, and amphotericin B, were purchased from Invitrogen (Carlsbad, CA, USA), as was Alexa-Fluor-conjugated secondary IgG antibody. Collagenase A was purchased from Roche Applied Science (Indianapolis, IN, USA). Recombinant human FGF-basic (basic-FGF) was purchased from Peprotech (London, UK). FNC coating mix (FNC) was purchased from Athena ES (Baltimore, MD, USA). Ascorbic acid, dimethyl sulfoxide (DMSO), Hoechst 33342 dye, methanol, mitomycin C, penicillin and streptomycin (P/S), Triton X-100, trypan blue, the MTT Cell Growth Assay Kit (MTT assay, CT02), and the ApopTag Plus In Situ Apoptosis Fluorescein Detection Kit (TUNEL assay, S7111) were purchased from Sigma-Aldrich (St. Louis, MO, USA). The Cellular ROS Assay Kit was purchased from Abcam (ab186029, Cambridge, MA, USA). Calcein-AM was purchased from BioVision, Inc. (Mountain View, CA, USA). Antibodies against Bcl2, p-Akt (Ser473), LC3, and Lamin A were purchased from Cell Signaling Technology (Beverly, MA, USA). p62 antibody was purchased from Abcam. Akt, β-actin, and GAPDH antibodies were purchased from Santa Cruz Biotechnology (Santa Cruz, CA, USA). All plastic cell culture dishes and plates were purchased from Corning Incorporated Life Sciences (Acton, MA, USA). 

### 2.2. Tissue Sources

All rabbits were purchased from registered farms and housed at the Animal Care Core Facility of Chang Gung Memorial Hospital in Linkou, Taiwan. All experimental and animal care procedures adhered to the Association for Research in Vision and Ophthalmology (ARVO) Statement for the Use of Animals in Ophthalmic and Vision Research. Corneal tissues were obtained from 4-month-old New Zealand white rabbits immediately after euthanasia, and the tissues were stored in 50 mL tubes containing DMEM for subsequent examination.

### 2.3. Cell Culture

The human retinal pigment epithelium cell line ARPE-19 (ATCC, Rockville, MD, USA) and the human corneal endothelial cell line B4G12 (Creative Bioarray, Shirley, NY, USA) were cultured in DMEM/F12 medium supplemented with 10% FBS and 1% P/S, or HESFM supplemented with 2% FBS and 10 ng/mL basic-FGF, respectively. The cells were maintained in a humidified incubator at 37 °C in an atmosphere of 5% CO_2_.

The procedures for isolation and culture of rabbit corneal endothelial cells (CECs) were modified from previous methods [12]. Briefly, Descemet’s membrane (DM; containing CECs) was stripped from the posterior surface of corneal tissues. The DM fragments were removed and digested at 37 °C for 16 h with 2 mg/mL collagenase A in Opti-MEM containing 50 μg/mL gentamicin and 5 μg/mL amphotericin B. After digestion, the CEC aggregates were collected by centrifugation and then cultured in 24-well plates coated with FNC. The CEC aggregates were maintained in RCEC medium (DMEM supplemented with 10% FBS and 25 μg/mL gentamicin) and subcultured for future investigation. 

### 2.4. Cell Viability

Cell viability was measured by MTT assay. In brief, the culture medium was removed and replaced with 100 µL of fresh medium. Next, 10 µL of the 12 mM MTT stock solution was added to each well, and the plates were incubated at 37 °C for 4 h. Finally, 100 µL of DMSO was added to each well, the well contents were mixed thoroughly by pipette, and the absorbance at 570 nm was read using a Sunrise ELISA reader (Tecan, Salzburg, Austria).

### 2.5. Reactive Oxygen Species (ROS) Detection

The cells were treated with the ROS inducer (paraquat) for 120 h, followed by incubation with detection reagents included in the Cellular ROS Detection Assay Kit for 30 min. Images were obtained using a Zeiss fluorescence microscope (Oberkochen, Germany). 

### 2.6. Apoptosis Detection 

The terminal deoxynucleotidyl transferase (TdT)-mediated dUTP-nick-end-labeling (TUNEL) technique was performed to detect apoptotic cells. After treatment with the ROS inducer (paraquat) for 120 h, cells were fixed with 1% paraformaldehyde and post-fixed with 2:1 ethanol:acetic acid for 5 min at −20 °C and then incubated with TUNEL reagents included in the ApopTag Plus In Situ Apoptosis Fluorescein Detection Kit for 1 h. After counterstaining with Hoechst 33342, cells were examined under a Zeiss fluorescence microscope.

### 2.7. Autophagosome Formation Detection

LC3 immunofluorescence was performed to detect autophagosome formation. After treatment with the ROS inducer (paraquat) for 120 h, cells were washed three times with PBS (pH 7.2), then fixed in 4% formaldehyde for 15 min at room temperature, rinsed with PBS, permeabilized with 0.2% Triton X-100 for 15 min, and then rinsed again with PBS. After incubation with 2% BSA to block non-specific staining for 30 min, the slides were incubated with the primary antibodies (LC3 at 1:100 dilution) for 24 h at 4 °C. After washing in PBS thrice, the slides were incubated with the corresponding Alexa Fluor-conjugated secondary IgG antibody (1:200 dilution) for 60 min at room temperature. The samples were then counterstained with Hoechst 33342 and examined under a Zeiss fluorescence microscope.

### 2.8. Western Blotting

Total cell lysates were prepared in RIPA buffer supplemented with 10 mmol/L sodium fluoride, 10 mmol/L sodium orthovanadate, and 1× protease inhibitor cocktail (Sigma-Aldrich). The suspensions were each transferred into a microfuge tube on ice, sonicated to disrupt the cells, and centrifuged for 15 min at 4 °C at maximum speed. The supernatants were then pooled to obtain the total protein extract. The protein extracts were resolved in 10% acrylamide gels and transferred onto polyvinylidene difluoride membranes (MilliporeSigma, Burlington, MA, USA), which were then blocked with 5% (*w*/*v*) fat-free milk in TBST (50 mmol/L Tris-HCl, pH 7.5, 150 mmol/L NaCl, 0.05% (*v*/*v*) Tween-20), and probed with the desired primary antibodies at 4 °C overnight, followed by reaction with appropriate horseradish peroxidase–conjugated secondary antibodies. The immunoreactive protein bands were visualized via enhanced chemiluminescence (GE Healthcare, Chalfont St Giles, UK).

### 2.9. Ex Vivo Rabbit Corneal Damage Model

The rabbit corneal tissues were cultured in tissue culture (TC) medium (DMEM supplemented with 10% FBS and 25 μg/mL gentamicin). The ascorbic acid-pretreatment groups were cultivated in TC medium containing 1 mM of ascorbic acid for two days, followed by the addition of paraquat (25 mM) in the paraquat-treatment groups for 15 min, and then washed with TC medium. Two days later, 1 μmol/L of Calcein-AM was added into TC medium for 15 min, followed by examination under a Zeiss fluorescence microscope to detect the sloughing status of corneal endothelial cells.

### 2.10. In Vivo Rabbit Corneal Damage Model

Rabbits received administration of 5% ascorbic acid (284 mmol/L in BSS solution) or BSS to the cornea three times a day for two days. Subsequently, 25 mM paraquat (diluted in BSS, total 20 mL) or BSS was slowly injected into the anterior chamber for 15 min using a syringe pump (Harvard Apparatus, Holliston, MA, USA). BSS infusion was performed for another 15 min for those with paraquat infusion. Afterwards, the topical administration of ascorbic acid three times a day was continued for two days. Corneal clarity was recorded using external eye photography. Following the sacrifice of the rabbits, corneal tissues and aqueous humor were obtained for further experiments.

### 2.11. Ascorbic Acid Measurement

Corneal tissues and aqueous humor were assayed using the OxiSelect™Ascorbic Acid Assay kit (FRASC, Cell Biolabs Inc., San Diego, CA, USA). Briefly, corneas were placed in 1× assay buffer, homogenized, and then centrifuged for 15 min at 4 °C at maximum speed. Subsequently, the samples, including corneal tissue extraction and aqueous humor, were mixed with an ascorbic acid standard and added into 96-well plates. Deionized water (−AO) or 1× ascorbate oxidase (+AO), together with the kit reaction reagents, were added into each well, followed by determination of optical densities at 540 nm. Finally, ascorbic acid concentration (μM) was calculated according to the standard curve.

### 2.12. Statistics

All data are presented as blots or images from at least three similar experiments or as mean ± S.D. for each group with at least three independent experiments performed. Student’s unpaired *t*-tests were performed using SPSS software version 13.0 (SPSS Inc. Chicago, IL, USA). Statistical significance is reported as two-tailed *p*-values, where *p* < 0.05 (*) and *p* < 0.01 (**) are considered statistically significant. 

## 3. Results

### 3.1. Ascorbic Acid Attenuates Oxidative Stress-Induced Cell Injury

Since phacoemulsification-induced damage with evenly distributed damage areas could not be reproduced for quantification in vitro, paraquat was chosen as an inducer of oxidative stress. In this study, the HCEC cell line (B4G12) was used as a cellular model to examine oxidative stress-induced damage in HCEC. In addition, the retinal pigment epithelium (RPE) cell line (ARPE-19) was used as a parallel control. It has been reported that damage caused by oxidative stress leads to apoptosis [27,28] and autophagic cell death [29] in RPE cells, which support normal functions in the retina.

To further investigate whether ascorbic acid protects cells from oxidative stress, paraquat (an inducer of ROS) was used at various concentrations (B4G12: 0, 0.1, 0.3 mM; ARPE-19: 0, 1, 3 mM). Subsequently, on day two, the viability of the B4G12 and ARPE-19 cells was quantified via MTT assay. Staurosporine, a non-inducer of ROS that incites cell toxicity by protein kinase inhibition, was used as a control. In contrast to the staurosporine group, pretreatment with ascorbic acid (0.25 and 1 mM) in the paraquat group was found to significantly protect cells in a dose-dependent manner (*p* < 0.01, Figure S1). 

**Figure 1:**
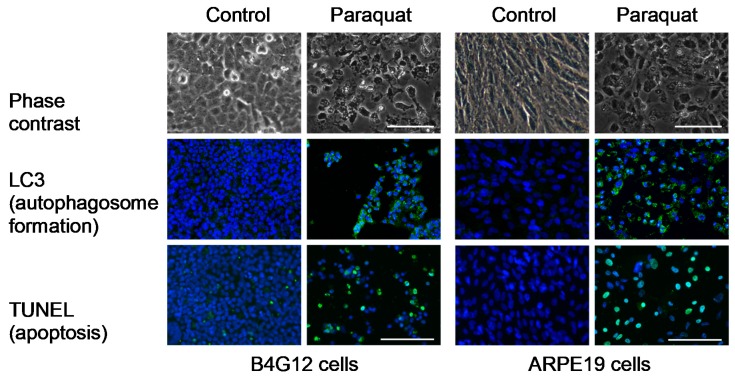
Oxidative stress induces apoptosis and autophagosome formation in B4G12 and ARPE-19 cells. The HCEC cell line (B4G12) and the RPE cell line (ARPE-19) were treated with paraquat (an oxidative stress inducer, 2 mM for ARPE-19, 0.2 mM for B4G12) for five days. Cell morphology was observed using phase-contrast microscopy. TUNEL assay and immunofluorescence of LC3 were performed to examine apoptosis and autophagosome formation (green color). Nuclei were counterstained with Hoechst 33342 (blue color). The scale bars represent 100 µm.

**Figure 2:**
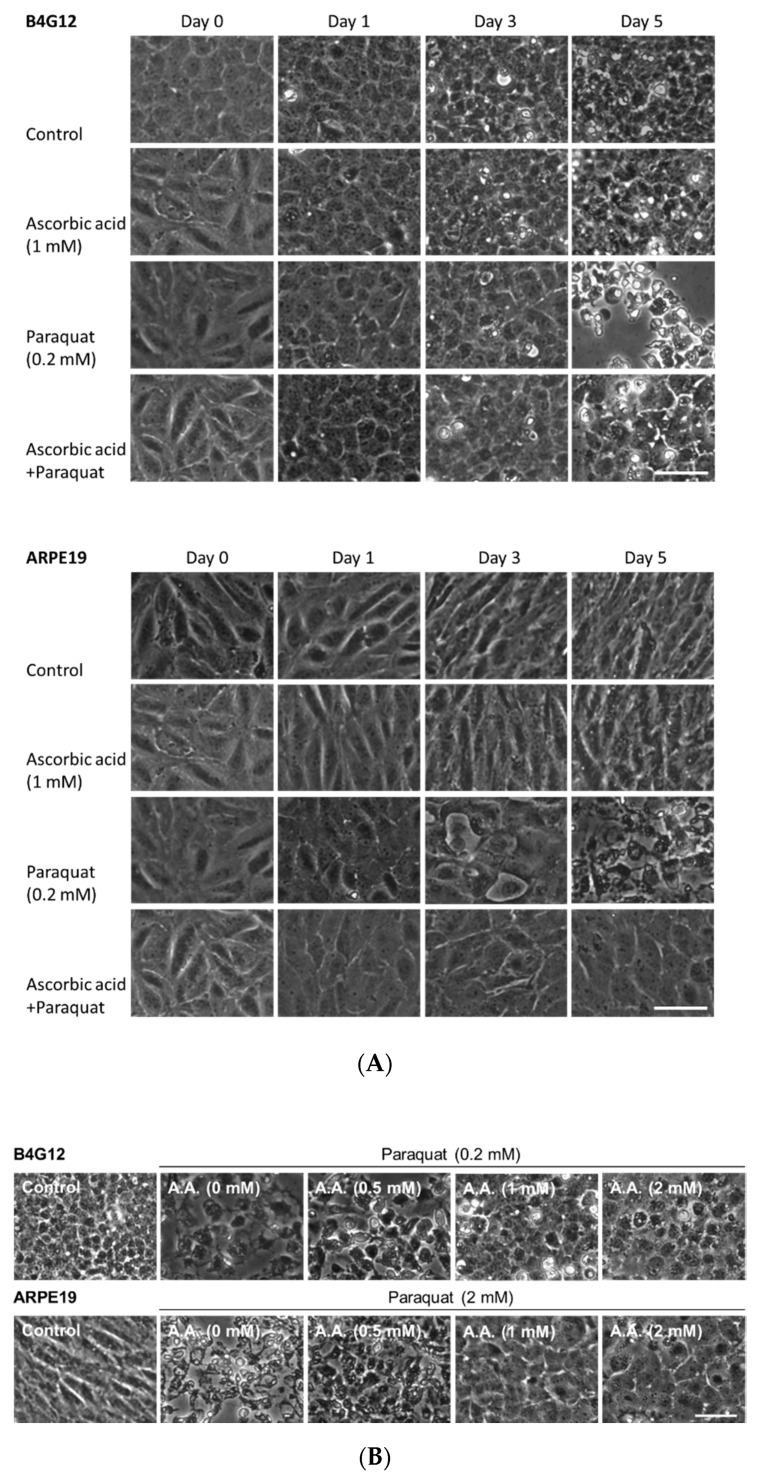
Ascorbic acid protects B4G12 and ARPE-19 cells from oxidative stress. B4G12 and ARPE-19 were pretreated with ascorbic acid (AA) for two days and further treated with paraquat (2 mM for ARPE-19, 0.2 mM for B4G12) for five days. (**A**) The time-dependent protective effect of ascorbic acid (1 mM) on B4G12 and ARPE-19 cells was examined on days 0–5. (**B**) The dose-dependent protective effect of ascorbic acid (0 to 2 mM) on B4G12 and ARPE-19 cells was examined on day five. Cell morphology was observed using phase-contrast microscopy, and images were captured at the same spot on different days. The scale bar represents 100 µm.

Cell apoptosis and autophagosome formation were examined via TUNEL assays and immunofluorescence staining for LC3. Whereas the control groups presented normal cellular structures, the paraquat-treated groups revealed cell loss, apoptosis, and autophagosome formation in both B4G12 and ARPE-19 cells on day five (Figure 1). We further examined the time- and dose-dependent protective effects of ascorbic acid by observing cell morphology. B4G12 and ARPE-19 cells were divided into four groups: control, AA (ascorbic acid), paraquat, and AA + paraquat. In the AA and AA + paraquat groups, cells were cultivated in medium containing 1 mM of ascorbic acid for two days. Treatment with paraquat (2 mM for ARPE-19, 0.2 mM for B4G12) was then performed in the paraquat and AA + paraquat groups. As shown in Figure 2A, cell morphology was observed on days 0–5. Compared to the control and AA groups, treatment with paraquat led to reduced cell density at day five, and this cell loss was rescued by pretreatment with 1 mM of ascorbic acid. As shown in Figure 2B, B4G12 and ARPE-19 cells were cultivated in medium containing 0, 0.25, 1, or 2 mM of ascorbic acid for two days, followed by the addition of paraquat (2 mM for ARPE-19, 0.2 mM for B4G12) for five days. According to morphological observations, ascorbic acid (1 and 2 mM) significantly rescued cell loss induced by paraquat. 

### 3.2. Ascorbic Acid Ameliorates Paraquat-Induced ROS and Oxidative Stress-Induced Apoptosis and Autophagic Flux Blockage in B4G12 and ARPE-19 Cells

**Figure 3:**
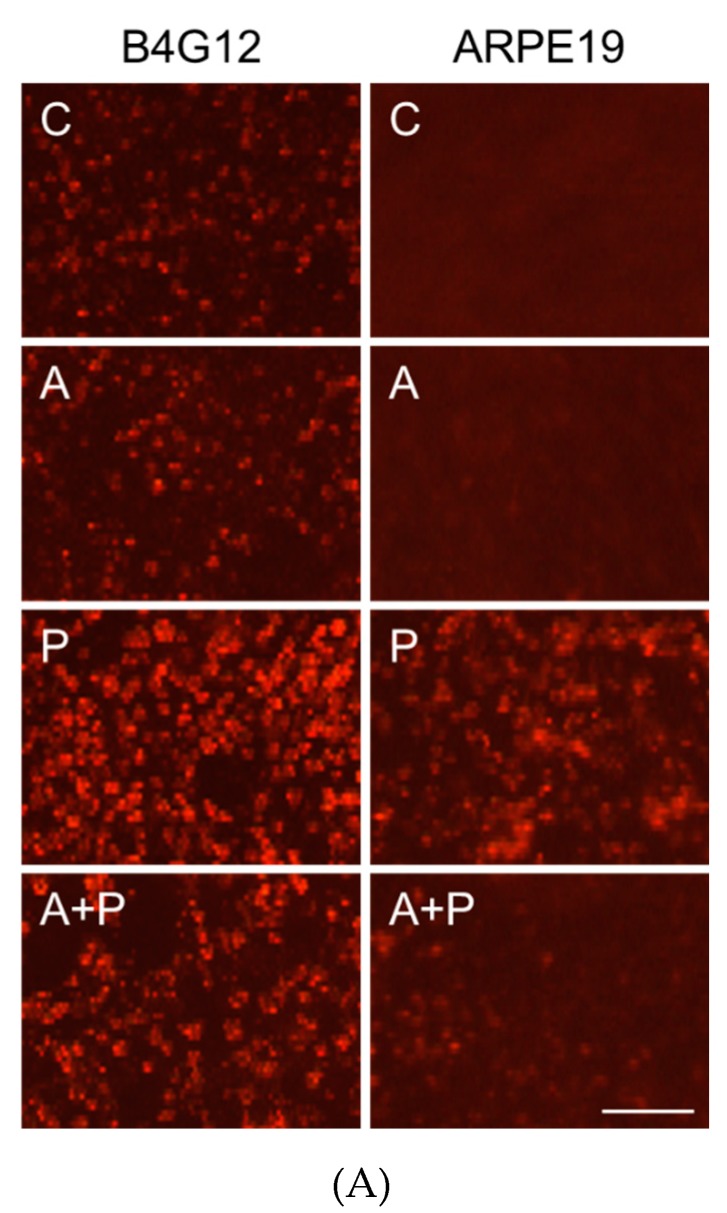

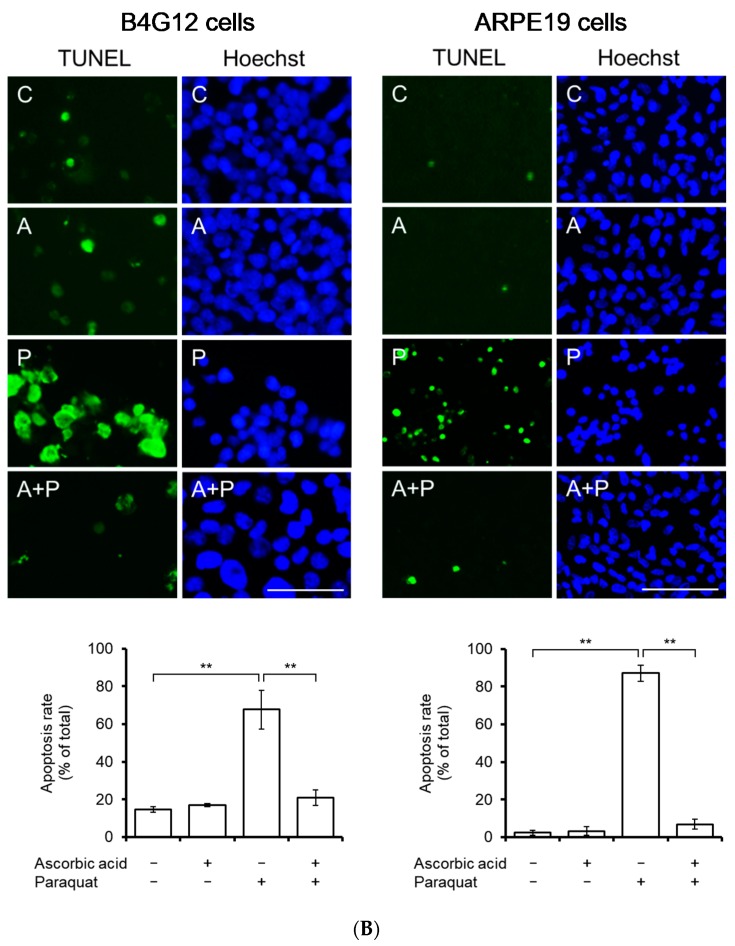

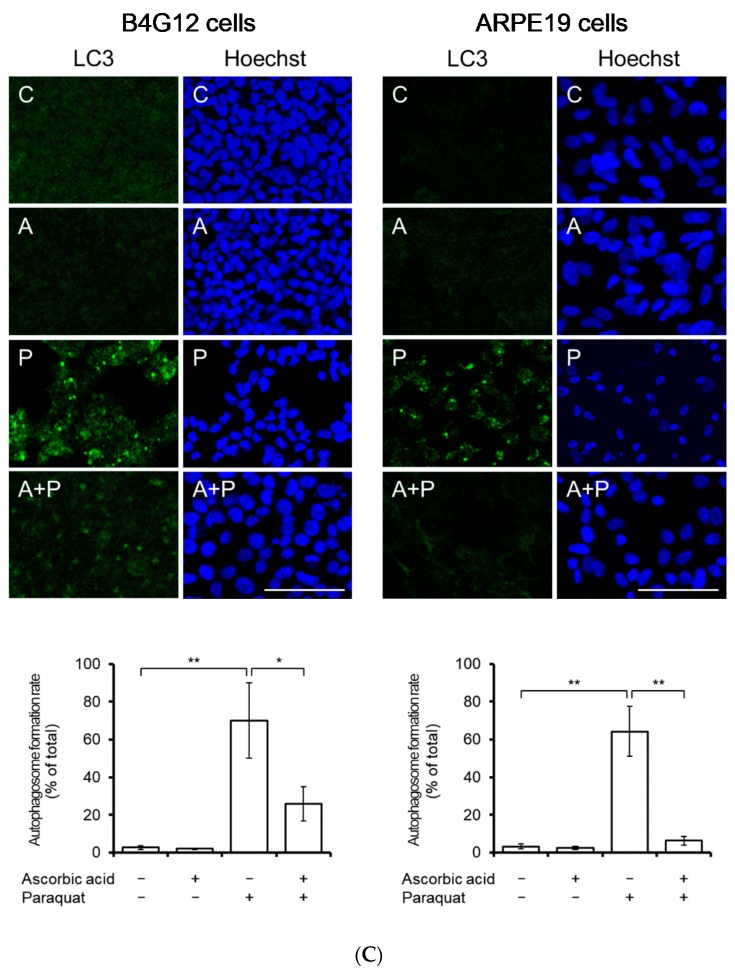

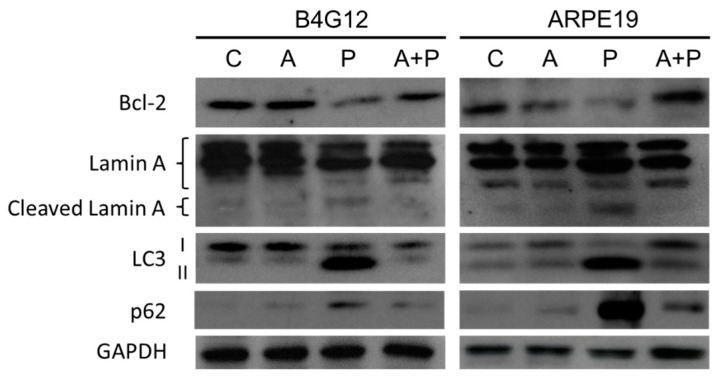

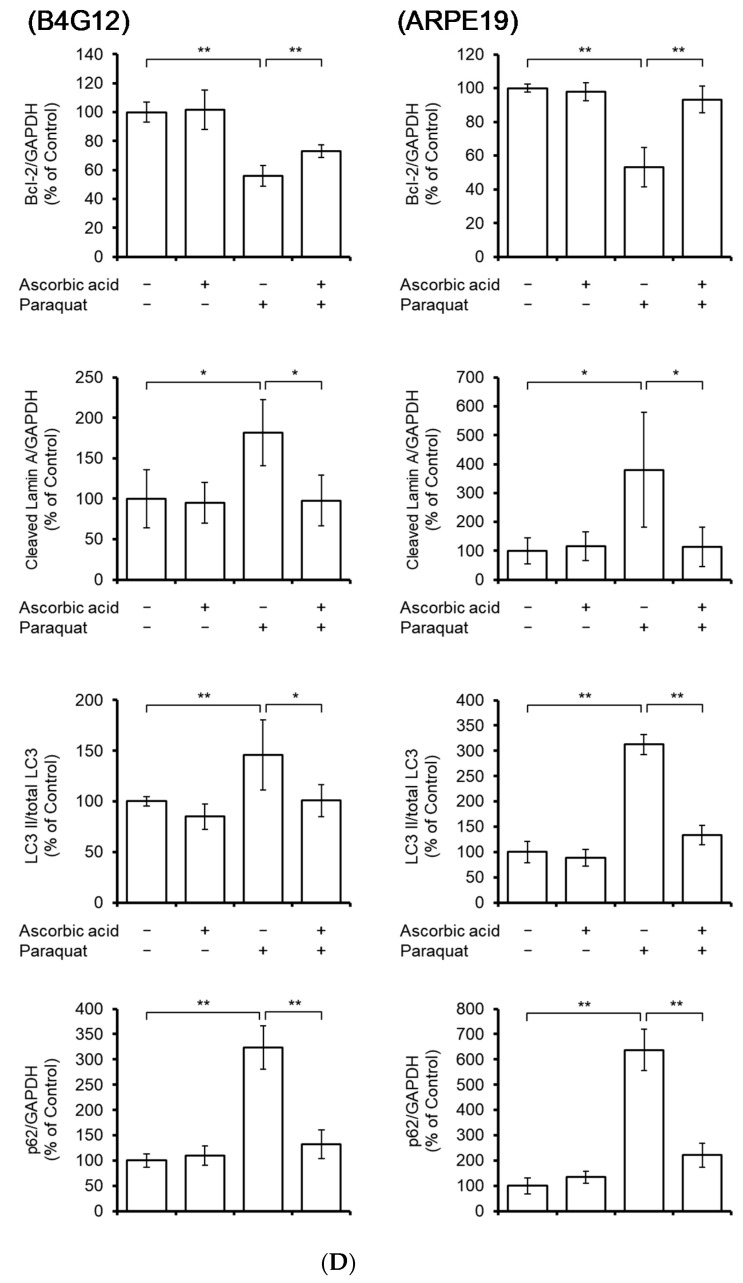
Pretreatment with ascorbic acid attenuates oxidative stress-induced apoptosis and autophagic flux blockage in B4G12 and ARPE-19 cells. B4G12 and ARPE-19 cells were cultivated in medium with or without 1 mM of ascorbic acid for two days, followed by the addition of paraquat (2 mM for ARPE-19 and 0.2 mM for B4G12) in the paraquat-treated groups (P only and C + P groups) for five days. (**A**) Paraquat-induced cellular accumulation of reactive oxygen species (ROS) and rescue by ascorbic acid was detected using ROS fluorescent dye (red color). ROS was induced by paraquat in both the B4G12 and ARPE-19 cells, and this was ameliorated by treatment with ascorbic acid. (**B**) Paraquat-induced apoptosis and rescue by ascorbic acid was examined using the TUNEL assay (green). (**C**) Paraquat-induced autophagosome formation and rescue by ascorbic acid was examined using immunofluorescence staining for LC3-II (autophagosome formation biomarker; green). (**D**) Effects of the paraquat and ascorbic on the protein expression of anti-apoptosis (Bcl-2), pro-apoptosis (lamin A, including cleaved forms), and autophagic flux (LC3 I/II and p62) biomarkers in B4G12 and ARPE-19 cells were probed using Western blotting. Paraquat induced altered protein expression in both B4G12 and ARPE-19 cells, and this could be reversed by ascorbic acid. The scale bars represent 100 µm (A) and 50 µm (B–C). Nuclei were counterstained with Hoechst 33342 (blue; B–D). (*n* = 3, * *p* < 0.05, ** *p* < 0.01).

Paraquat-induced ROS was detected using ROS fluorescent dye. Paraquat treatment (2 mM for ARPE-19, 0.2 mM for B4G12) significantly increased cellular ROS in both B4G12 and ARPE-19 cells. However, pretreatment with 1.0 mM ascorbic acid significantly rescued paraquat-induced ROS (Figure 3A). Paraquat treatment (2 mM for ARPE-19, 0.2 mM for B4G12) significantly increased apoptosis (B4G12, 14.6% ± 1.4% vs. 67.7% ± 10.2% *p* < 0.01; ARPE-19, 2.4% ± 1.3% vs 87.1% ± 4.4%, *p* < 0.01) and autophagosome formation (B4G12, 2.7% ± 1.0% vs. 70.2% ± 19.9%, *p* < 0.01; ARPE-19, 3.2% ± 1.3% vs. 64.2% ± 13.2%, *p* < 0.01) in the B4G12 and ARPE-19 cells. Likewise, pretreatment with 1.0 mM ascorbic acid significantly attenuated paraquat-induced apoptosis (B4G12, 67.7% ± 10.2% vs. 20.9% ± 4.1% *p* < 0.01; ARPE-19, 87.1% ± 4.4% vs. 6.9% ± 2.6% *p* < 0.01) and autophagosome formation (B4G12, 70.2% ± 19.9% vs. 25.8% ± 9.1% *p* < 0.01; ARPE-19, 64.2% ± 13.2% vs. 6.4% ± 2.3% *p* < 0.01) in both the B4G12 and ARPE-19 cells after five days in culture (Figure 3B,C). 

To validate the results of the immunofluorescence assay, an immunoblotting assay was also performed. Bcl2 has previously been regarded as an anti-apoptotic protein, while lamin A cleavage has been considered a marker of apoptosis in the cornea [30]. Recently, between the two forms of LC3, LC3-I, and LC3-II, the autophagosome membrane-bound form (LC3-II) has been viewed as a marker of autophagosome formation. During the process of autophagy, p62 binds to LC3-II to facilitate degradation of ubiquitinated protein aggregates [31] and degrades in autolysosome. Therefore, the protein level of LC3-II and p62 could be regarded as an index of autophagic flux change. As shown in Figure 3D, paraquat treatment significantly suppressed the expression of Bcl2 protein, whereas ascorbic acid in the medium significantly enhanced the expression of Bcl2. In contrast, paraquat treatment significantly elevated the expression of cleaved lamin A, LC3-II and p62, whereas ascorbic acid in the medium significantly suppressed the expression of cleaved lamin A, LC3-II, and p62 in both ARPE-19 and B4G12 cells.

**Figure 4:**
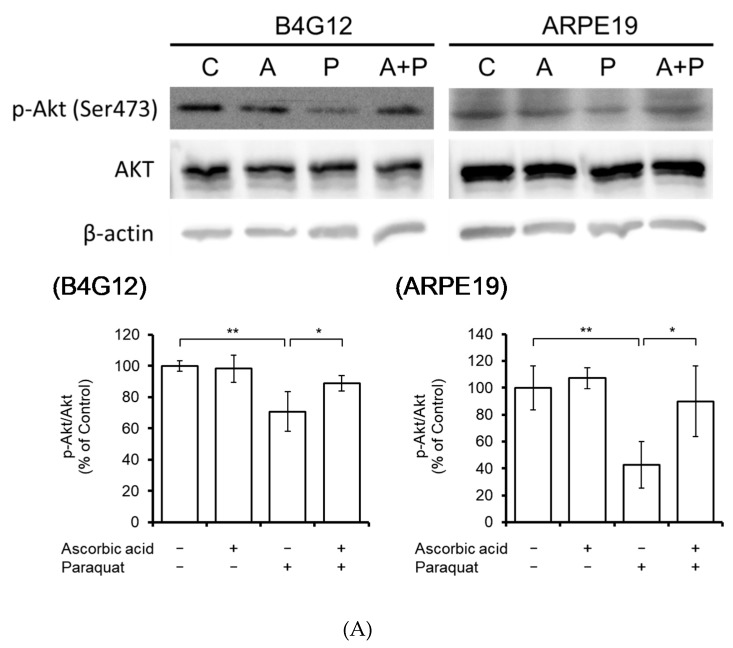

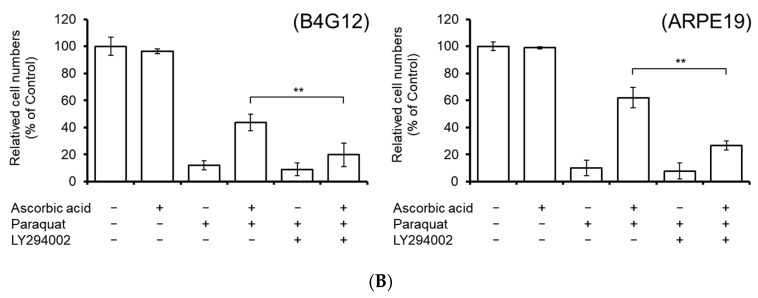
The PI3K/AKT pathway is involved in ascorbic acid-mediated cell protection. (**A**) Phosphorylation of Akt (Bcl-2 upstream regulator) was detected by Western blotting. Total Akt and β-actin were used as loading controls. Paraquat-suppressed Akt phosphorylation was rescued by ascorbic acid. (**B**) To examine whether the PI3K/AKT pathway was involved in ascorbic acid-mediated cell protection, LY294002 (a PI3K inhibitor, 50 μmol/L) was added in the culture medium. The cell protection effect was quantified by cell counting. Paraquat-induced cell loss was rescued by pretreatment with ascorbic acid, while the rescue effect of cell loss by ascorbic acid was significantly negated by the addition of LY294002. (*n* = 3, * *p* < 0.05, ** *p* < 0.01).

Given that phosphorylation of Akt (Ser473) is a known upstream activator of Bcl-2 and that the PI3K/Akt pathway is involved in ROS-induced autophagic cell death in human CECs [32,33], we investigated the changes in Akt phosphorylation. As shown in Figure 4A, paraquat treatment significantly reduced Akt phosphorylation (p-Akt/Akt), while the addition of ascorbic acid resulted in the reversal of Akt phosphorylation. To further verify the involvement of the PI3K/Akt pathway in the ascorbic acid-mediated protection from ROS-induced cell death, we added LY294002 (PI3K inhibitor, 50 μmol/L) to the medium and observed that this significantly diminished the protective effect (reduced cell loss) of ascorbic acid in B4G12 and ARPE-19 cells (Figure 4B).

### 3.3. Topical Ascorbic Acid Ameliorates Oxidative Stress-Induced Corneal Endothelial Damage in Rabbits

To investigate whether topical ascorbic acid protects CEC against oxidative stress, we established an in vivo rabbit model of corneal endothelial damage. Accordingly, we first examined the protective effect of pretreatment with ascorbic acid on the primary rabbit corneal endothelial cells (RCECs) in both in vitro and in vivo culture models.

**Figure 5:**
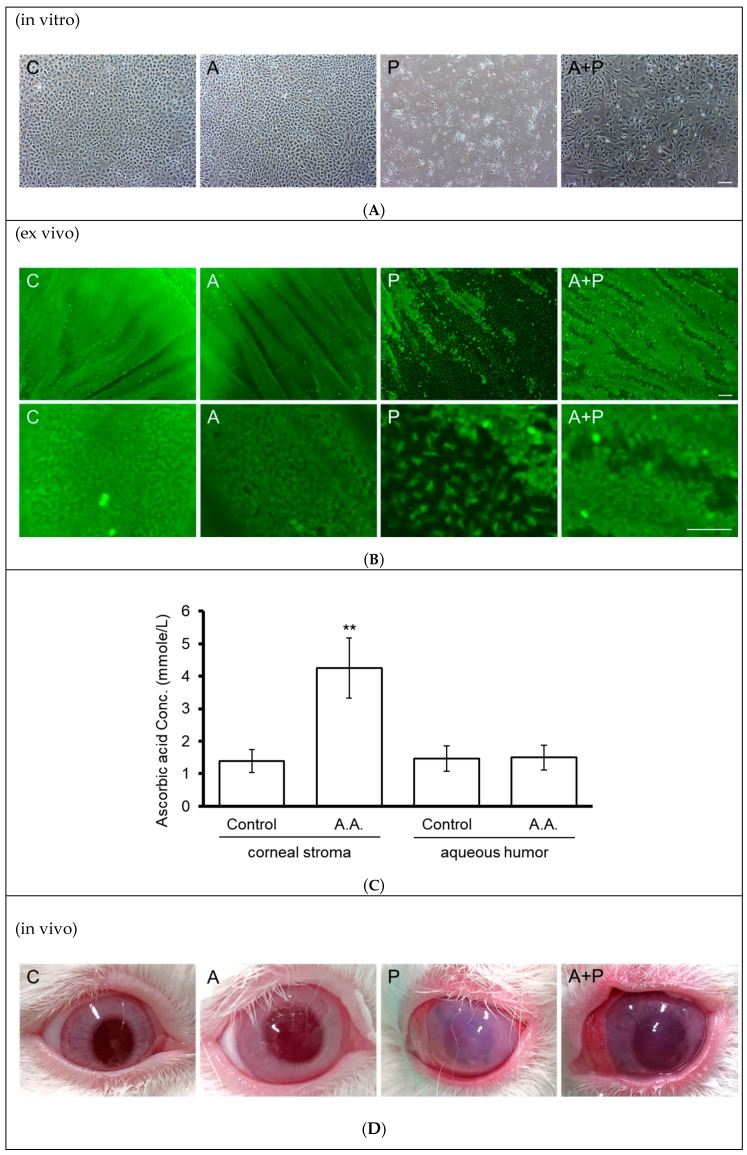
Effect of topical ascorbic acid on oxidative stress-induced corneal endothelial damage in a rabbit model. (**A**) Primary rabbit corneal endothelial cells (in vitro) were cultivated in medium with (A and A + P groups) or without (C and P groups) addition of 1 mM of ascorbic acid for two days, followed by addition of paraquat (0.2 mM) in the paraquat-treated groups (P and A + P groups) for five days. Sloughing of corneal endothelial cells was observed under phase-contrast microscopy. (**B**) Rabbit corneal tissue specimens (ex vivo) were cultivated in medium with (A and A + P groups) or without 1 mM of ascorbic acid for two days, followed by the addition of paraquat (25 mM) to the P and A + P groups for 15 min. Two days later, the sloughing of corneal endothelial cells was observed using Calcein-AM stain. (**C**) Rabbit corneas (in vivo) received an application of ascorbic acid (284 mmol/L in BSS solution) or BSS alone for two days (three times per day). After diffusion, ascorbic acid concentrations in the corneal stroma and anterior chambers were examined using the FRASC assay. (*n* = 3, ** *p* < 0.01). (**D**) Rabbit corneas (in vivo) received an application of ascorbic acid (284 mmol/L in BSS solution) or BSS alone for two days (three times per day), followed by intracameral injection of 25 mM paraquat (diluted in BSS) or BSS alone for 15 min. Corneal transparency was assessed using external eye photography on day two. (The scale bar represents 100 µm).

In the in vitro cell culture, confluent primary RCECs (passage 1) were divided into four groups. The A (ascorbic acid) and A + P (ascorbic acid and paraquat) groups were cultivated in medium containing 1 mM of ascorbic acid (A) for two days. This was followed by the addition of 0.2 mM of paraquat in the P (paraquat) and A + P groups for five days. Similar to the results in B4G12 and ARPE-19 cells, paraquat induced cell toxicity in RCECs, while pretreatment with ascorbic acid protected against cell loss (Figure 5A).

It is difficult to continuously maintain an elevated paraquat concentration in the anterior chamber in the animal model. This is because systemic toxicity, such as hepatorenal dysfunction, develops in two to three days at a low, poisonous dose of paraquat. Therefore, we chose to establish a model of corneal endothelial damage induced by perfusion of high concentration (25 mmol/L) of paraquat over a short time period (15 min). Hence, we performed ex vivo corneal tissue culture. As in the in vitro experiments, rabbit corneal tissues were divided into four groups. The A and A + P groups were cultivated in medium containing 1 mM of ascorbic acid for two days, followed by the addition of 25 mM of paraquat for the P and A + P groups for 15 min. Two days later, the extent of CEC sloughing was observed using Calcein-AM stain. As shown in Figure 5B, paraquat treatment resulted in the sloughing of RCECs, while pretreatment with ascorbic acid ameliorated loss of RCECs.

Subsequently, after two consecutive days of topical application of ascorbic acid (284 mmol/L) three times a day, diffusion of ascorbic acid into the corneal stroma and anterior chamber was recorded. As shown in Figure 5C, the ascorbic acid concentration was significantly elevated in the corneal stroma (1.39 ± 0.35 vs. 4.24 ± 0.92 mmol/L, *p* < 0.01), but showed no difference in the aqueous humor (1.46 ± 0.39 vs. 1.50 ± 0.38 mmol/L).

Finally, external eye photography was used to monitor the transparency of the cornea in the in vivo rabbit model. CEC damage induced by infusion of paraquat (25 mmol/L) for 15 min resulted in diminished corneal transparency. In contrast, the topical application of ascorbic acid for two consecutive days prior to induction of damage using paraquat improved corneal transparency (Figure 5D).

## 4. Discussion

Corneal endothelial cell density (ECD) tends to be decreased by phacoemulsification-induced oxidative stress. It has been demonstrated in a canine model that intracameral infusion of ascorbic acid during phacoemulsification minimizes the loss of corneal endothelial cells [34]. Nevertheless, the protective effect of topically administered ascorbic acid on the corneal endothelium has not yet been defined. Clinically, a combined protocol of topical ascorbic acid and steroids has been adopted to treat alkali-induced eye injuries to achieve rapid wound healing and prevent severe ocular sequelae [35]. Recently, we have reported successful phacoemulsification using topical ascorbic acid perioperatively in two patients with low corneal ECD [36]. In the current study, we investigate whether topical ascorbic acid protects HCEC against cell death induced by external oxidative stress.

Phacoemulsification is not the only risk factor for elevated levels of oxidative stress in the anterior chamber. Direct exposure to environmental and solar UV radiation introduces alternative sources of oxidative stress in the cornea, such as photo-oxidative damage [37]. In the corneal endothelium, oxidative stress decreases the levels of cytochrome oxidase and antioxidants; meanwhile, it increases lipid peroxidation, leading to cellular impairment of HCEC [38,39]. In fact, Fuchs endothelial corneal dystrophy (FECD) is characterized by prominent apoptosis resulting from excessive oxidative stress and oxygen-induced DNA damage [39,40]. Hence, it is plausible that topical ascorbic acid could serve a protective role in patient populations with vulnerable corneal endothelium. 

As shown in Figure 5C, AA concentration in the corneal stroma significantly increases after a topical application of AA. According to Fernández-Pérez J, AA induces the dendritic morphology, increases the expression of keratocyte markers, and prevents myofibroblast differentiation [41]. Therefore, topical use of AA is not likely to produce side effects on keratocytes. On the other hand, why the AA concentration is not different in the aqueous humor is probably because the aqueous circulation neutralizes the changing pattern of AA concentration. In contrast, there is no dynamic circulation in the interstitial fluid within the corneal stroma, and a long-term reservoir effect can be formed. In addition, Descemet’s membrane is AA-penetrable, so that AA from the corneal stroma can exert effects on the corneal endothelium, explaining our observation of improved corneal transparency after topical use of AA (Figure 5D). 

In the current study, we have demonstrated that paraquat-provoked oxidative stress induces apoptosis and autophagic flux blockage in corneal endothelial cells, and that inhibition of the PI3K/Akt pathway attenuates the protective effects of ascorbic acid (Figure 4). In addition to the direct reduction of ROS by ascorbic acid, alleviating apoptosis and autophagic flux blockage, the PI3K/Akt pathway has also been known to suppress apoptosis through activation of Bcl-2 [42]. Moreover, previous evidence has shown an autophagosome formation -inhibitory effect of activation of the PI3K/AKT pathway [43]. It remains unclear whether or not the aforementioned pathways are involved in the protective effect of ascorbic acid.

When cells are under oxidative stress, p62/SQSTM1 is induced to express and creates a positive feedback loop by inducing antioxidant response element-driven gene transcription [44]. However, when autophagic defect (blockage of autophagic flux) occurs, the autophagosome fails to fusion with the lysosome, resulting in the accumulation of p62 [45]. Accumulation of p62 subsequently provokes autophagic defect-dependent apoptosis by activating caspase 8 [46,47]. In Figure 3D, paraquat treatment significantly increased the level of LC3-II, indicating an increased formation of the autophagosome. On the other hand, paraquat increases the level of p62, suggestive of autophagic defect induced by paraquat. AA treatment significantly decreased the level of p62, indicating that reducing autophagic defect might be one of the candidate pathways of AA protecting cells from oxidative stress-induced apoptosis. Moreover, crosstalks between autophagy and apoptosis have been shown to regulate cellular survival or death under stress [48]. Therefore, the autophagy-mediated survival pathway could pave the way for therapeutic strategies, for which further investigation is warranted.

As one of the important anti-oxidative constituents in the anterior chamber, levels of ascorbic acid decline as a result of the aging process [49]. In this study, we confirm that the topical administration of ascorbic acid leads to an elevated concentration in the corneal stroma, which subsequently enhances its antioxidant activity against paraquat-induced oxidative stress. Therefore, we propose that topically applied ascorbic acid results in a preventive effect against oxidative stress-associated ocular degeneration.

## Supplementary Materials

The following are available online at https://www.mdpi.com/2073-4409/9/4/943/s1, Figure S1: Ascorbic acid improved cell viability from oxidative stress.
