# Supplementary material for: Topical Ascorbic Acid Ameliorates Oxidative Stress-Induced Corneal Endothelial Damage via Suppression of Apoptosis and Autophagic Flux Blockage
Source: Cells. 2020 Apr 11;9(4):943. doi: 10.3390/cells9040943 (PMC7227019; doi:10.3390/cells9040943)

Figure S1. Ascorbic acid improved cell viability from oxidative stress

B4G12 and ARPE19 were cultivated in the medium containing different concentrations of ascorbic acid (0 mM to 1 mM) for two days, and then further treated with different concentrations of cytotoxicants including paraquat (oxidative stress inducer, 0 mM to 3 mM for ARPE-19 and 0.1 mM to 0.3 mM for B4G12) and staurosporine (protein kinase inhibitor, 0 mM to 1.6 mM) for two days. The MTT assay was used to quantify cell viability. Compared to that in the staurosporine group, cell viability in the paraquat group was significantly rescued in a dose-dependent manner. (* $P < 0.05$, ** $P < 0.01$)

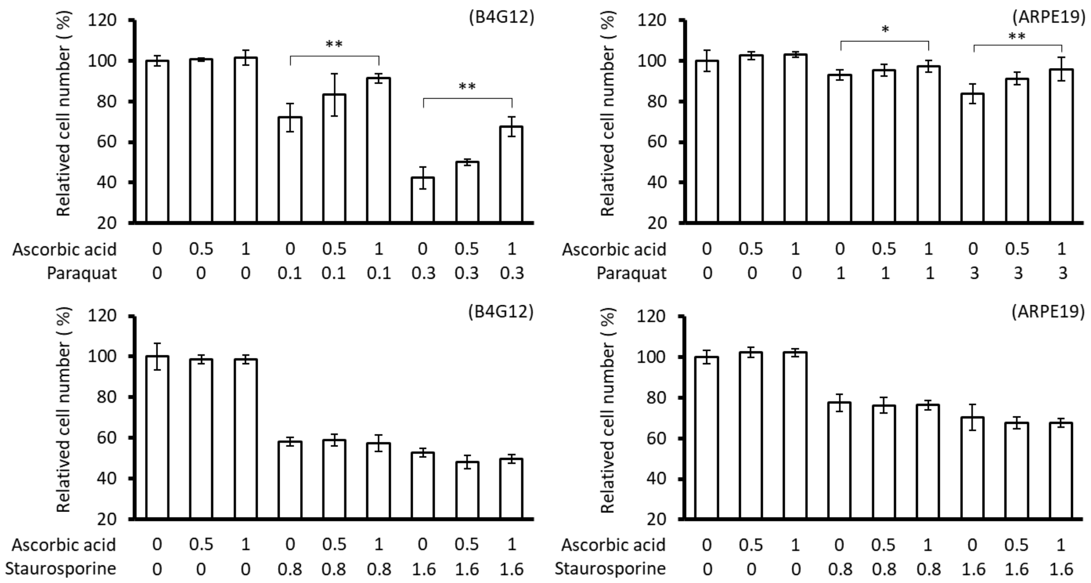

Supplement: Supplementary file 1 [file cells-09-00943-s001.pdf]
